# Bumetanide Therapeutic Effect in Children and Adolescents With Autism Spectrum Disorder: A Review Study

**DOI:** 10.32598/bcn.9.10.380

**Published:** 2019-09-01

**Authors:** Raheleh Mollajani, Mohamad Taghi Joghataei, Mehdi Tehrani-doost

**Affiliations:** 1. Cognitive Neuroscience Institute for Cognitive Science Studies, Tehran, Iran.; 2. Department of Anatomy and Neuroscience, Cellular and Molecular Research Center, Iran University of Medical Sciences, Tehran, Iran.; 3. Research Center for Cognitive and Behavioral Sciences, Tehran university of Medial Sciences, Tehran, Iran.

**Keywords:** Bumetanide, Diuretics, Autism Spectrum Disorder (ASD)

## Abstract

**Introduction:**

Autism Spectrum Disorder (ASD) is characterized by several impairments in communications and social interactions, as well as restricted interests or stereotyped behaviors. Interventions applied for this disorder are based on multi-modal approaches, including pharmacotherapy. No definitive cure or medication has been introduced so far; therefore, researchers still investigate potential drugs for treating ASD. One of the new medications introduced for this purpose is bumetanide. The present article aimed to review the efficacy of this drug on the core symptoms of ASD and its potential side effects.

**Methods:**

We searched all papers reported on pharmacokinetics, pharmacodynamics, efficacy, and adverse effects of bumetanide on animal models and humans with ASD. The papers were extracted from the main databases of PubMed, Web of Science, and Scopus.

**Results:**

The findings revealed that cortical neurons have high Chloride ion (Cl−)i and excitatory actions of gamma-aminobutyric acid in the valproic acid animal model with ASD and mice with fragile X syndrome. Bumetanide, which has been introduced as a diuretic, is also a high-affinity-specific Na+−K+−Cl− cotransporter (NKCC1) antagonist that can reduce Cl− level. The results also indicate that bumetanide can attenuate behavioral features of autism in both animal and human models. Moreover, the studies showed that such medication could activate fusiform face area in individuals with ASD while viewing emotional faces. Also, recent findings suggest that a dose of 1 mg/d of this drug, taken twice daily, might be the best compromise between safety and efficacy.

**Conclusion:**

Recent studies provided some evidence that bumetanide can be a novel pharmacological agent in treating core symptoms of ASD. Future studies are required to confirm the efficacy of this medication in individuals with ASD.

## Highlights

- An imbalance in excitatory/inhibitory functions in the early development of the brain can be an etiological factor in developing autism spectrum disorder.
- In the early development of the brain, GABA acts as an excitatory agent, which leads to the accumulation of the chloride ions inside the cell.
- Bumetanide, a loop diuretic, is an antagonist to the NKCC1 receptor and can reduce the intracellular chloride ion and shift GABA from excitation to inhibition state.
- Several studies have shown that bumetanide can reduce the symptoms of the autism spectrum disorder and can be considered a new treatment for this disorder.

## Plain Language Summary

Autism Spectrum Disorder (ASD) is a neurodevelopmental disorder characterized by impairments in fulfilling social relationships with others, along with repetitive behaviors and interests. The prevalence rate of ASD has increased in recent years. The main treatments used for this disorder have been non-pharmacological. However, a few approved medications such as risperidone and aripiprazole could reduce some non-core symptoms of ASD, but no use for improving social interactions in these patients. Recently, bumetanide has been introduced as a new medication to improve the core symptoms of ASD. Bumetanide has been already utilized as a diuretic for several years. Some studies have also reported the efficacy of this medication in improving symptoms of children with ASD, such as social relationships. The present article reviewed the studies reporting efficacy and adverse effects of bumetanide in patients with ASD.

## 1. Introduction

Autism is a neurodevelopmental disorder in children, characterized by impairments in communications and social interactions, as well as limited and repetitive patterns of behaviors and interests. A recent investigation reported that 1 in 88 children and 1 in 54 boys in America have autism (Prevalence of Autism Spectrum Disorders, 2012). To date, the United States Food and Drug Administration and European Medicines Agency have not approved any drugs and effective medical treatments for improving the core symptoms of Autism Spectrum Disorder (ASD). However, two authorized medicines to treat autism-related irritability are risperidone (for 5–16 years old children) and aripiprazole (for 6–17 years old children).

Both risperidone, a dopamine type 2 (D2) and serotonin type 2A (5-HT2A) receptor antagonist (McCracken, 2002), and aripiprazole, a partial dopamine (DA) D2 and serotonin 1A receptor (5-HT1A) agonist and a 5-HT2A antagonist (Wink, Erickson, & McDougle, 2010) have failed to treat the core symptoms of ASD (Lemonnier et al., 2017). Also, both have been found to show some side effects, including sedation, vomiting, extrapyramidal syndromes, increased appetite, drowsiness, drooling, and weight gaining (Fung et al., 2016; Lemonnier et al., 2017).

Bumetanide, a diuretic and chloride cotransporter antagonist, has been recently proposed as a new therapeutic strategy (Lemonnier et al., 2012; Lemonnier & Ben-Ari, 2010). A wealth of evidence demonstrates the level of chloride and GABAergic signaling change in animal models of ASD, patients with ASD, and other developmental disorders (Ben-Ari, 2015).

Bumetanide has been widely used in adults since 1975 and children from 1986 for the treatment of acute and chronic disorders such as hypertension, nephritic syndrome, blockade-associated heart failure, and dysplasia-associated bronchopulmonary. The drug can restore low Cl−i level and shift GABA from excitation to inhibition; therefore, it can also be used for a wide range of disorders (Ben-Ari, 2015; Blaesse, Airaksinen, Rivera, 2009; Nardou et al., 2011). This article aimed to search various studies on bumetanide and autism together and investigate the efficacy of this drug in behavioral and cognitive symptoms of ASD.

## 2. Methods

The databases of Web of Science, PubMed, and Scopus were searched with the following keywords: “ASD” , “Autism”, “Asperger”, “Pervasive Developmental Disorder”, and “Bumetanide.” Moreover, we searched for the pharmacological functions of bumetanide through those mentioned databases. Since the papers in this field are limited, we did not use any strategy for excluding the articles.

## 3. Pharmacological Characteristics of Bumetanide

### 3.1. Chemistry

**Figure 1.**
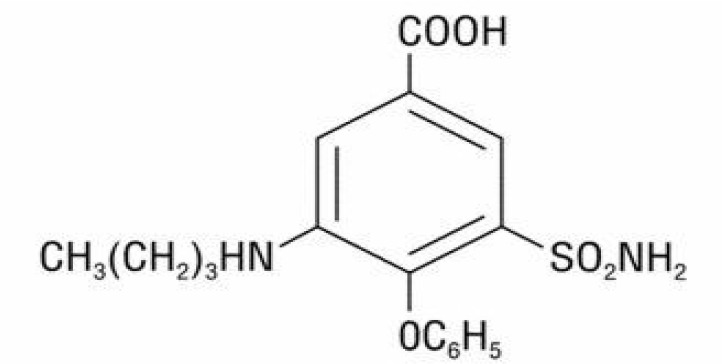
The structural formula of bumetanide

Bumetanide, a newly emerged diuretic agent, has the same properties of other diuretics affecting the loop, such as furosemide and ethacrynic acid (Dixon et al., 1976). It is a 4-substituted derivative of sulfamoylbenzoic acid with the chemical name of 3-N-butylamino-4-phenoxy-5-sulfamoylbenzoic acid. Replacing the chlorine atom with a phenoxy group also discriminate bumetanide from other sulfamoyl diuretics (Figure 1) (Turmen, Thom, Phillip, 1982).

### 3.2. Pharmacokinetics

Bumetanide has a short half-life (between 90 min and 3 h) and can barely pass through the blood-brain barrier (Li et al., 2011). Following oral administration of 2.0 mg of bumetanide to healthy volunteers, these general characteristics are observed: approximately full absorption; a peak plasma level at 30 minutes of roughly 80 ng/mL; an obvious half-life of 1.2–1.5 hours; and an average plasma elimination rate of approximately 228–255 mL/min (Dixon et al., 1976; Pentikäinen, Neuvonen, Kekki, 1980). The clearance of bumetanide is triple, with a half-life ranging from 6 minutes to 3 hours. It can also be rapidly and almost eliminated by urinary excretion and metabolism (Dixon et al., 1976). The metabolism of bumetanide occurs through the butyl side chain, and alcohol is its primary metabolite (Flamenbaum & Friedman, 1982).

### 3.3. Pharmacodynamics

#### 3.3.1. Gama-aminobutyric acid and autism spectrum disorder

The exact molecular mechanisms by which ASD acts are not fully elucidated; however, several new studies have indicated several clinical pictures of autism are due to activity-related dysregulation of neural development. ASD has also been linked to mutations that occur in some voltage-gated and ligand-gated ion channels, which are essential in excitability regulation of neurons and calcium ion (C2+) signaling pathway (Krey & Dolmetsch, 2007). Anomalies in neuronal excitability during development can also occur due to changes in neurotransmitter systems (Barnby et al., 2005). Among such systems, some evidence supports the deficiency of GABAergic inhibition in autism (Krey & Dolmetsch, 2007; Menold et al., 2001). Patients with autism are often recognized by rearrangements in chromosome 15q11-13, constructing a cluster of genes related to Gama-Aminobutyric Acid (GABA) genes, including GABRA5, ABRG3, and GABRB3 (Dykens, Sutcliffe, & Levitt, 2004; Menold et al., 2001).

Polymorphisms in GABRA4 are also associated with autism (Collins et al., 2006; Ma et al., 2005). Likewise, other genes that are important in the differentiation and migration of GABAergic interneurons and regulate the development of GABAergic neurons such as ARX are related to ASD. ARX mutations can also result in epilepsy, movement disorders, cortical malformations, mental retardation, and autism (Friocourt, Poirier, & Rakić, 2006; Turner, Partington, & Kerr, 2002). Nevertheless, GABA is considered as a major excitatory neurotransmitter during early development resulting in depolarization of neurons and Ca^2+^ influx through voltage-gated Ca^2+^ channels. Such GABA-recruited Ca^2+^ transmissions may be critical in a large number of neurodevelopmental aspects, including proliferation, migration, dendritic arborization, and development of Purkinje cell (Represa & Ben-Ari, 2005).

Provocation of GABA receptors is also crucial in the production of simultaneous concurrent network function and modulation of Ca^2+^ waves in cortex development (Garaschuk, Linn, & Eilers, 2000; Voigt, Opitz, & De Lima, 2001). Therefore, a deficit in the function of GABA can lead to disturbance in the developing brain, which may cause some neurodevelopmental disorders such as ASD.

#### 3.3.2. Bumetanide and Gama-aminobutyric acid

As previously mentioned, GABA, as a neurotransmitter inhibitor in an adult brain, acts as a stimulant in the early stages of the postpartum period. This mechanism is based on the accumulation of chloride inside the cell and a “reversed” chloride gradient in a wide variety of neurons and animal species, especially invertebrates (Ye, 2004)

Moreover, extensive studies have shown that the two chloride co-transporters of NKCC1 and KCC2 play an essential role in this growing trend. NKCC1 and KCC2 are respectively considered as the most critical chloride importers and exporters in a way that intracellular chloride levels are mainly controlled by them (Ben-Ari, Gaiarsa, & Tyzio, 2007; Ben-Ari, 2014; Lemonnier et al., 2012). The high-affinity-specific NKCC1 antagonist can reduce Cl−i and shift GABA from excitation to inhibition (Blaesse et al., 2009; Nardou et al., 2011). It is notable that oxytocin, which improves social communications (Guastella, Mitchell, & Dadds, 2008), like bumetanide acts by decreasing Cl−i levels (Tyzio et al., 2006).

#### 3.3.3. Bumetanide and autism spectrum disorder

According to the reviewed studies (Table 1), maternal infusion of bumetanide in the ASD animal model and mice with fragile X syndrome reduced the physiological levels of Cl−i in offspring and consequently regulated electrical and behavioral parameters (Eftekhari et al., 2014; Tyzio et al., 2014). The animal studies also showed that GABAergic signals had changed in mice suffering from fragile X syndrome (Chao et al., 2010; Coghlan et al., 2012; Pizzarelli & Cherubini, 2011; Tabuchi et al., 2007). Recent measurements of intracellular chloride in neurons of mice with fragile X syndrome from birth to adulthood also demonstrate increased levels associated with GABA-stimulating activity (Tyzio et al., 2014).

**Table 1.** Behavioral and cognitive effects of Bumetanide studies in Autism Spectrum Disorder (ASD)

**Study**	**Participants**	**Method: (Design, Diagnostic Measures, Intervention, Duration)**	**Outcome**
Tabuchi et al., 2007	Neuroligin-3 R451C KI and neuroligin-3 KO mice	An animal study. Quantitative Western blots, whole-cell recordings of layer 2/3 of the somatosensory (barrel) cortex in acute slices, dark/light box, novel home cage activity, open field arena, elevated plus maze, and Morris water maze.	A significant elevation in the expression of two markers for inhibitory synapses (the vesicular GABA-transporter VGAT and the postsynaptic protein gephyrin) in the R451C KI mice, while no difference was observed in VGAT expressions in the KO mice. These data vigorously demonstrated that a difference in the inhibitory/excitatory balance might participate in the pathogenesis of ASDs.
Chao et al. 2010	Mice with MeCP2 deficiency in GABAergic neurons (male Viaat-Mecp22/y mice and male littermate controls)	An animal study. RT–qPCR, Immunolabelling, mIPSCs, a partition test, and modified three-chamber assay, Morris water maze, EEG.	MeCP2 deficiency found in GABAergic neurons led to diminished presynaptic function of GABA release and revealed a multitude of neuropsychiatric phenotypes. Findings indicated that GABAergic dysfunction is a crucial cause of Rett syndrome and autistic phenotypes.
Pizzarelli and Cherubini, 2011	ASD and fragile X animal models	Review; Focuses on the implications of altered GABAergic signaling in neurodevelopmental disorders such as ASDs	Lower frequency of GABAergic interneuron networks in the cortex, olfactory bulb, and hippocampus, as well as in the GABA-mediated tonic inhibition. Dysfunction of the GABAergic signaling early in development and a severe E/I unbalance in neuronal circuits, which can account for some of the behavioral deficits observed in ASD patients.
Coghlan et al., 2012	Animal models of ASDs and related disorders	Review of evidence from genetics, molecular neurobiology, and systems neuroscience relating to the role of GABA in ASD and associated diseases, including fragile X syndrome, Rett syndrome, and Fetal Anticonvulsant Syndrome.	The results showed that some of the subunits of the GABAA receptor system have functional roles in neurodevelopment, and there is a GABA deficit in autism, fragile X syndrome, and Rett syndrome.
Tyzio et al., 2014	Two animal models of autism: rats exposed to valproate in the utero (VPA rats) and mice with the fragile X mutation (FRX mice).	An experimental study. Bumetanide (10 μM) or oxytocin (1 μM). Whole-cell voltage-clamp recordings, isolation-induced ultrasonic vocalizations, Intracranial EEG recordings. Bumetanide (10 μM) or oxytocin (1 μM)	Acute applications of Bumetanide significantly decreased (Cl–)i in neurons observed in VPA rats and FRX mice. KCC2 was down-regulated in the hippocampi of VPA rats and FRX mice. Maternal pretreatment with Bumetanide restored electrophysiological and behavioral phenotypes and blocking oxytocin signaling, produced autistic-like electrophysiology and behavior in offspring.
Eftekhari et al., 2014	VPA rats and FRX mice.	An experimental study Acute applications of bumetanide (10 μM) or oxytocin (1 μM) (Tyzio et al., 2014). The social approach-avoidance paradigm, three-chamber social test.	Adult male VPA rats that treated with bumetanide before birth (maternal pretreatment) displayed improved sociability than age-matched non-treated VPA rats. Adult male FRX mice indicated a significantly higher number of grooming events (bouts) than wild-Type (WT) itermates. Bumetanide treatment around delivery attenuated autistic behavioral features in adult offspring.
Lemonnier and Ben-Ari, 2010	Five neonates with autism.	A pilot study. ICD-10 criteria. Five standard IAS severity tests, ADI-R, CARS, ABC, CGI, RDEG, and RRB. Bumetanide (1 mg/24 hours or 0.5 mg/twice a day). Three months.	Bumetanide improved the behavioral aspects of IAS. The study reported a significant improvement in IAS with no side effects.
Lemonnier et al., 2012	Sixty children with autism or Asperger syndrome (3–11 years old) in two groups.	A double-blind, randomized, placebo-controlled trial. CARS, CGI, ADOS G, Placebo or bumetanide (1 mg daily). Three months.	Bumetanide significantly reduced CARS, CGI, and ADOS Values. Side effects were limited to mild hypokalemia. Bumetanide improved the symptoms of ASD and is a promising novel therapeutic agent to treat autism.
Hadjikhani et al., 2013	Seven high-functioning males with ASD.	An open trial pilot study. DSM-IV-TR. ADOS, ADI-R the Autism- Spectrum Quotient (AQ), Empathy Quotient (EQ), WASI, and fMRI. Bumetanide treatment (1 mg/d). Ten months.	Improvement of emotional face recognition and activation of brain regions were involved. Areas included rewards, motivations, and emotions also showed an increase in activity.
Lemonnier et al., 2013	A 10-year-old boy with fragile X.	A single case report. CARS, ADOS, ABC, RDEG, and RRB. Bumetanide treatment twice a day (0.5 mg morning and 0.5 mg evening). Three months.	Decrease the intensity of autistic symptoms. The only side effect was mild hypokalemia. The results raised the possibility of treating FRX children with bumetanide with a good benefit/risk ratio.
**Bruining et al., 2015**	A 10-year-old girl with ASD, dysplasia of the cortex, and duplication in 15q11.2 locus.	A single case report. Repetitive Behavior Scale-Revised, BRIEF, The digit span of the WISC–III and The spatial span of the Wechsler Nonverbal Scale of Ability, The Rey Auditory Verbal Learning Test, The Rey Visual Design Learning Test, The Amsterdam Neuropsychological Tasks battery, and EEG recordings. Bumetanide treatment (0.5 mg/twice daily). Six months.	A specific improvement in sensory behaviors, rigidity, learning, and memory performance. Bumetanide improved neural functioning, which supported by changes in resting-state EEG. The power of α frequency elevated following the treatment that may explain another mechanism contributing to the improvement of the patients in behavioral and cognitive functions.
Du et al., 2015	Sixty children with autism (two categories: Single treatment and combined treatment categories.	A pilot study. ICD-10 criteria. ABC, CARS, SI, GI, CGI. Bumetanide treatment (0.5 mg BID) and ABA training. Three months.	The total score of ABC, CARS, and SI were reduced in both groups. The overall scores of ABC and CGI in the combined treatment category were significantly reduced in comparison to the single treatment category. CARS in the combined treatment category was lower than the treatment category but not significantly. No undesirable effects were found.
Lemonnier et al., 2017	Eighty-eight patients with ASD (2–18 years old). Groups received Bumetanide (0.5, 1.0, or 2.0 mg twice daily) or placebo.	A double-blind, randomized, placebo-controlled, multisite dose-ranging study. CARS, SRS, CGI-I. Three months.	The most common adverse effects were as follows: Hypokalemia, increased urine elimination, loss of appetite, dehydration, and asthenia. The prevalence and incidence of adverse effects directly correlated with the dose of bumetanide. Bumetanide made better ASD core symptoms and exhibited a favorable beneficial/detrimental ratio, especially at 1.0 mg BID.

ASD: Autism Spectrum Disorder; BID: Bis In Die (twice a day); GABA: Gamma-Aminobutyric Acid; IAS: Infantile Autistic Syndrome; CARS: Childhood Autism Rating Scale; ABC: Aberrant Behavior Checklist; CGI: Clinical Global Impressions; RRB: Repetitive and Restrictive Behavior; RDEG: Regulation Disorder Evaluation Grid; ADOS: Autism Diagnostic Observation Schedule.

Based on these observations, the effects of bumetanide on five children with autism were evaluated using 5 standard tests of Infantile Autistic Syndrome (IAS) severity tests, including Childhood Autism Rating Scale (CARS), Aberrant Behavior Checklist (ABC), Clinical Global Impressions (CGI), Repetitive and Restrictive Behavior (RRB), and Regulation Disorder Evaluation Grid (RDEG) (Lemonnier & Ben-Ari, 2010).

In this study, the diuretic was used (1 mg/d as 0.5 mg BID), and treatment continued for 3 months, and the results suggested that bumetanide improved behavioral aspects of IAS with no side effects. Lemonnier et al., in a randomized, double-blind study on 60 children aged 3–11 years with autism or Asperger syndrome receiving placebo or bumetanide (1 mg/d for 3 months) found that it had significantly reduced CARS, CGI, and Autism Diagnostic Observation Schedule (ADOS) values. The side effects were also limited to mild hypokalemia treated with supplemental potassium. The results confirmed the earlier small-scale open-label pilot study in 2010 and showed that bumetanide had ameliorated ASD symptoms (Lemonnier et al., 2012).

Also, an open-trial pilot study on seven adolescents and young adults with autism assessing the effect of 10 months of treatment with bumetanide showed an improvement of facial emotion recognition and activation of brain regions involved, including the inferior occipital cortex and the fusiform gyrus. Increased activity in areas that are responsible for rewards, motivations, and emotions, including nucleus accumbens, amygdala, and cortex orbitofrontal, was suggested, as well. These results support the benefits of bumetanide as a promising treatment to ameliorate social interactions in autism (Hadjikhani et al., 2013). The results of a single case report in 2013 using CARS, ADOS, ABC, RDEG, and RRB, before and after treatment, showed that daily use of bumetanide for 3 months in a 10-year-old boy with fragile X syndrome had decreased the intensity of ASD symptoms. As reported in this study, the only side effect was mild hypokalemia (Lemonnier et al., 2013).

In 2015, Bruining reported a 10-year-old girl with ASD, dysplasia of the cortex, and duplication in 15q11.2 locus; meanwhile, she had epilepsy. She indicated extreme excitement in her behaviors following the previous treatment with Clavazam (a benzodiazepine). In this respect, the treatment led to a specific improvement in sensory behaviors, rigidity, and memory performance (Bruining et al., 2015).

In a pilot study using bumetanide in combination with education (0.5 mg BID) on 60 children with autism indicated that the total score of ABC, CARS, and SI (severity of disease) reduced in both groups after 3 months compared with their scores at the pre-treatment stage. The overall scores of ABC and CGI in the combined treatment group were significantly lower than the single treatment one. Although the total scores and CARS-related item scores in the group of combined treatment were less than the treatment group after a 3-month intervention, there were no significant results. Generally, treatment with bumetanide and ABA training compared with the only ABA training led to more improvements in children with autism. No undesirable effects of bumetanide were observed (Du et al., 2015).

## 4. Effective Dose and Side Effects of Bumetanide

In a recent study to determine the best dose of bumetanide in the pediatric population (Lemonnier et al., 2017) the NKCC1 chloride-importer inhibitor bumetanide restores physiological (Cl-, the results showed that the medication was safe with some side effects associated with diuresis and dehydration. Lemonnier et al. also used bumetanide twice a day, and the results indicated that the frequency and severity of side effects increased with dose, while there was no clear relationship between dose and efficacy. According to the results of this study, the dose of 1 mg twice a day seemed to be the best balance between safety and efficacy.

As mentioned above and based on the results of most studies, administration of bumetanide was safe and had some minor adverse effects, which were mainly limited to hypokalemia (Hadjikhani et al., 2013; Lemonnier et al., 2012, 2017; Lemonnier et al., 2013; Lemonnier & Ben-Ari, 2010) the NKCC1 chloride-importer inhibitor bumetanide restores physiological (Cl-. This adverse effect can be controlled by regular monitoring of the kalemia and kidney function in the patients (Hadjikhani et al., 2013).

Hypokalemia was also clinically manageable with potassium supplementation, dose stabilization, and precise hydration of patients (Lemonnier et al., 2017) the NKCC1 chloride-importer inhibitor bumetanide restores physiological (Cl-. In other words, the changes observed after taking bumetanide (and other potent diuretics) are hypokalemia, hypochloremia, hypochloremic metabolic alkalosis, and hyperuricemia (without gastro-esophageal arthritis). It should be noted that biochemical tests commonly used for drug safety did not show any important disturbances other than transitory thrombocytopenia or granulocytopenia (Flamenbaum & Friedman, 1982).

## 5. Conclusion

ASD is known as a neurodevelopmental disorder characterized by an obvious excitatory/inhibitory imbalance in selective neuronal circuits, particularly in the GABAergic signaling. Likewise, numerous studies have indicated that drugs that affect GABAergic synapses can ameliorate behavioral defects in animal models of autism and improve at least some of the symptoms observed in ASD patients. Experimental works and clinical observations have further shown that bumetanide as a safe loop diuretic (Lemonnier et al., 2017) the NKCC1 chloride-importer inhibitor bumetanide restores physiological (Cl-, may be a potential treatment strategy that improves ASD symptoms without serious adverse effects. However, future clinical trials are needed to confirm the efficacy of bumetanide in ASD.

## Ethical Considerations

### Compliance with ethical guidelines

Since this is a review paper, there were no ethical issues apart from considering the rights of the authors.
